# High-throughput human primary cell-based airway model for evaluating influenza, coronavirus, or other respiratory viruses in vitro

**DOI:** 10.1038/s41598-021-94095-7

**Published:** 2021-07-22

**Authors:** A. L. Gard, R. J. Luu, C. R. Miller, R. Maloney, B. P. Cain, E. E. Marr, D. M. Burns, R. Gaibler, T. J. Mulhern, C. A. Wong, J. Alladina, J. R. Coppeta, P. Liu, J. P. Wang, H. Azizgolshani, R. Fennell Fezzie, J. L. Balestrini, B. C. Isenberg, B. D. Medoff, R. W. Finberg, J. T. Borenstein

**Affiliations:** 1 Bioengineering Division, Draper, Cambridge, MA 02139 USA; 2 Department of Medicine, University of Massachusetts Medical School, Worcester, MA USA; 3 Division of Pulmonary and Critical Care Medicine, Massachusetts General Hospital, Boston, MA USA

**Keywords:** Viral infection, Biomedical engineering

## Abstract

Influenza and other respiratory viruses present a significant threat to public health, national security, and the world economy, and can lead to the emergence of global pandemics such as from COVID-19. A barrier to the development of effective therapeutics is the absence of a robust and predictive preclinical model, with most studies relying on a combination of in vitro screening with immortalized cell lines and low-throughput animal models. Here, we integrate human primary airway epithelial cells into a custom-engineered 96-device platform (PREDICT96-ALI) in which tissues are cultured in an array of microchannel-based culture chambers at an air–liquid interface, in a configuration compatible with high resolution in-situ imaging and real-time sensing. We apply this platform to influenza A virus and coronavirus infections, evaluating viral infection kinetics and antiviral agent dosing across multiple strains and donor populations of human primary cells. Human coronaviruses HCoV-NL63 and SARS-CoV-2 enter host cells via ACE2 and utilize the protease TMPRSS2 for spike protein priming, and we confirm their expression, demonstrate infection across a range of multiplicities of infection, and evaluate the efficacy of camostat mesylate, a known inhibitor of HCoV-NL63 infection. This new capability can be used to address a major gap in the rapid assessment of therapeutic efficacy of small molecules and antiviral agents against influenza and other respiratory viruses including coronaviruses.

## Introduction

The specter of a pandemic respiratory virus such as influenza or the current outbreak of SARS-CoV-2 represents one of the greatest threats to human health in modern history^1,2^. For influenza, the development of efficacious, long-lasting, and seasonally-consistent vaccinations has been challenged by the occurrence of both antigenic drift and shift^3^. Currently, four antivirals are in wide use for treatment of influenza, three neuraminidase inhibitors and more recently, a cap-dependent endonuclease inhibitor, baloxavir marboxil^4^. These treatments are generally effective, but genetic changes to endemic viruses present challenges similar to those encountered during the development of vaccines^5^. These challenges are exacerbated by the long developmental timelines typically associated with manufacturing, scale up, and regulatory approval for novel antivirals or vaccines. The COVID-19 pandemic has spurred aggressive vaccine development efforts, but even with this accelerated timeline, the current wave of infection is projected to yield significant morbidity and mortality^6^. Therefore, a critical element of medical treatment strategy is the application of FDA-approved antiviral medications originally developed for other diseases^7^.

Several preclinical research tools are available to model respiratory viruses, but due to both the complexity of the human lung and the need to accurately model species-specific viral infections, these systems are limited in their ability to serve as a basis for guiding clinical strategy^8^. For example, a standard approach used to investigate influenza A viruses (IAV) typically begins with the inoculation of immortalized cell lines in conventional culture well plates, such as Madin-Darby canine kidney (MDCK) or Calu-3 cells^9,10^. While these cells infect easily and provide a rapid early assessment of the potential efficacy of a therapeutic for IAV, they lack the critical mechanisms and signaling pathways that healthy human airway tissues provide in response to viral infection. Therefore, although these systems are easy to use, they are not often accurate predictors of human clinical efficacy^11^. For coronaviruses, Calu-3 or Vero E6 cells are often used for similar reasons, but again differ by species, cell source, disease state, and innate antiviral signaling pathways^12,13^ compared to normal, healthy human conducting airway and alveolar epithelium^14^, thus providing an insufficient and unreliable model. Unfortunately, challenges also exist with animal models of IAV, not only in mice, rats, and guinea pigs, but even in more human-relevant options for evaluation, such as ferrets and macaques^15,16^. These models often require species-adapted viral strains, and fail to recapitulate features of human innate antiviral responses and clinical signs of infection^8,17^. Similar challenges exist for coronavirus infection studies, where the low-throughput and limited availability of appropriate animal models, combined with concerns about their predictive power, force early and often high-risk testing in humans. While many experimental studies are underway to identify drugs with antiviral activity against SARS-CoV-2^18^, the discovery pipeline would benefit significantly from rapid and accurate preclinical models based on human primary cell culture.

Challenges with preclinical models and tools have spurred the development of cell culture platforms comprising human primary cells in a microfluidic environment, commonly known as microphysiological systems or organs-on-chips^19,20^. Over the past two decades, these devices have evolved from early toxicology platforms^21^ to today’s wide range of technologies including multi-organ systems^22–24^ and a variety of organ-specific models including airway or alveolar tissue systems^25,26^. While the integration of human primary cells or stem cell-derived populations in a physiologically-relevant microenvironment represents a tremendous advance over the use of immortalized cell lines in principle, in practice the application of these systems in pharmacological research and development has been gated by several factors. These limitations include low-throughput (ranging from single microchamber devices to 6 – 24-device configurations) and complex operation, a lack of relevant metrics for system assessment, the use of research-grade materials and components for platform construction, the lack of critical aspects of tissue or organ structure required to replicate infection, and most critically, an absence of confidence in the in vitro*-*in vivo correlation (IVIVC) for these technologies.

Our group has previously reported on systems that offer higher throughput, compatibility with existing pharmaceutical laboratory infrastructure, convenient readouts for quality control and physiological monitoring, and applicability for preclinical studies across many disease areas and application domains^27,28^. These platforms represent a significant advance over current organ-on-chip systems in several ways, including increased throughput (96 independent microtissues), dynamic perfusion of each independent chamber, real-time sensing of barrier function, compatibility with in-situ imaging during operation of the plate, and a reduced media volume that minimizes utilization of cells and reagents for operational efficiency. Here we build on that work from our group^28^ and the work of others^29^ to establish an in vitro model with an ALI capability sufficient to support physiologically relevant epithelial differentiation such as that in airway, gut or kidney models. For functional proximal airway or alveolar models, human primary cells should be cultured at an ALI, representing a suitable analogue for the respiratory barrier tissues that mimics mucus formation, mucociliary flow, and a range of physiologically relevant responses^25,26,30^. Our airway model provides this ALI and supports IAV and coronavirus infection, while enabling high-throughput, real-time studies across various viral strains, multiplicities of infection (MOI), human donors, time points, and statistically significant numbers of replicates on a single 96-device engineered platform. Viral infection kinetics are evaluated for two strains of IAV, A/California/04/09 H1N1, and A/Hong Kong/8/68 H3N2, as well as the response of infection with the A/Hong Kong/8/68 H3N2 strain to dosing with oseltamivir, demonstrating application of the system toward evaluation of efficacy of antiviral therapeutic compounds.

Currently, in vitro systems for evaluation of therapeutics for COVID-19 rely upon high throughput well plate-based systems cultured with immortalized cell lines such as Vero E6, Calu-3 and A549. More recent developments include the use of genetic modification techniques to confer physiologically relevant properties to various immortalized cell lines, induced pluripotent stem cell (iPSc)–based systems, and the development of human primary epithelial cell-based technologies including organoids and various lung on a chip systems. While these latter approaches provide a more physiologically relevant microenvironment for investigating key mechanisms of SARS-CoV-2 infection, they are generally limited in throughput and in the ability to fully integrate into the laboratory infrastructure and pharmaceutical development pipelines. Here we report on the first application of a high throughput organ-on-chip system capable of evaluating coronavirus infection across multiple viral strains and human primary cell donors on a single plate. First we confirm expression of the receptor ACE2 and the serine protease TMPRSS2 in human primary airway epithelial tissues cultured in the PREDICT96-ALI system, a key attribute toward modeling infection with HCoV-NL63 and SARS-CoV-2. We demonstrate viral propagation and infection kinetics of HCoV-NL63, which, like SARS-CoV and SARS-CoV-2, utilizes ACE2 as its target receptor. We also evaluate HCoV-OC43 infection in the system across MOI and human donors, providing evidence of the extensibility of the system. Finally, as a proof-of-concept demonstration toward future use of the system in evaluation of therapeutic efficacy of SARS-CoV-2 in the model, we present data on the treatment of HCoV-NL63-infected tissues with camostat mesylate, a protease inhibitor that blocks coronavirus entry.

## Methods

### Microfluidic platform

The PREDICT96 organ-on-chip platform consists of a microfluidic culture plate with 96 individual microchannel-based culture chambers (or devices) and a perfusion system driven by 192 microfluidic pumps integrated into the plate lid^28^. The standard configuration comprises individual pumps independently addressing the apical and basal chambers of each of the 96 devices on the platform. Here the platform is adapted to support culture of barrier tissues, such as airway tissues, by establishment and maintenance of an ALI, where only the basal compartments are pumped with circulating media.

**Figure 1:**
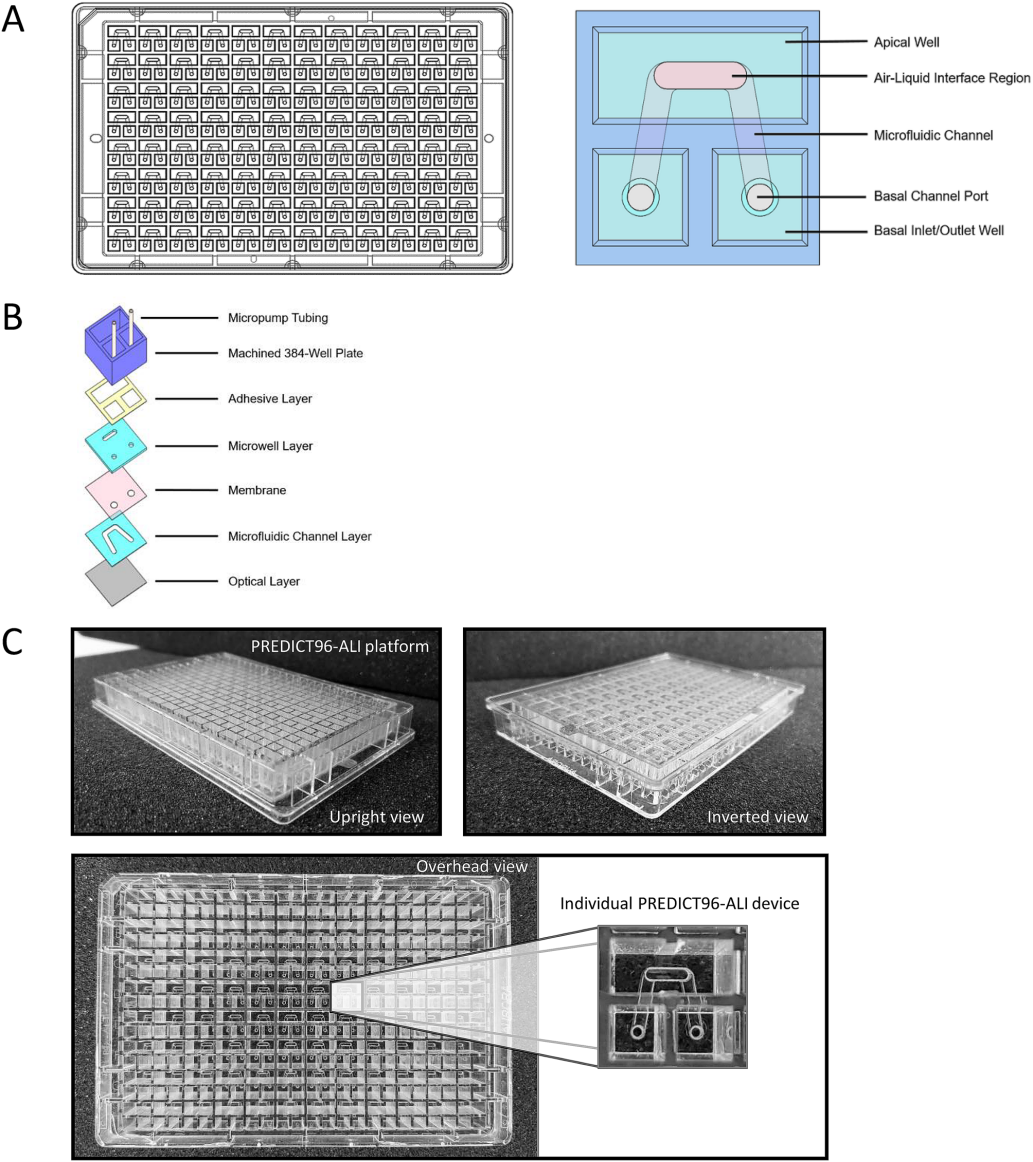
PREDICT96-ALI platform. (**A**) Schematics illustrating the configuration of the PREDICT96-ALI platform, with the overall standard 384-well plate layout shown at the left. Detail for an individual airway model element of the array is shown at right, where the top chamber appears as an oval region and the underlying bottom chamber an inverse U-shaped channel with inlet and outlet ports. (**B**) Exploded view stack of patterned polymer films used to create the structure, which is fabricated at the plate level but is shown at the level of an individual model. (**C**) Photographs of the upright view (left), inverted view (right) and overhead view (bottom), with magnified view of an individual model within the PREDICT96-ALI platform configuration.

Each device in the PREDICT96-ALI culture plate was formed from a 2 × 2 array within a standard 384-well plate, with the wall between the top two wells removed to create a 3-well cluster. This configuration is shown in Fig. 1A. A slotted hard plastic microwell (0.864 mm height, 1 mm width, 2.5 mm length) was positioned in the center of the large well, with a microfluidic channel (0.25 mm height, 1 mm width) aligned underneath that was linked to the two remaining wells in each culture chamber. The channel was capped from below with a thin optically clear layer. A microporous membrane was used to separate the top microwell chamber from the bottom microchannel culture chamber to permit the establishment of an ALI.

To fabricate the PREDICT96-ALI culture plate, a UV laser system (Protolaser U4: LPKF Laser and Electronics, Garbsen, Germany) was used to laser-cut thin films of cyclic olefin polymer (COP) (ZF14-188: Zeon Corp., Tokyo, Japan) and cyclic olefin copolymer (8007 COC: Tekni-plex, Wayne, PA, USA). The COP layers were adhered together using low-glass transition temperature COC in a heated hydraulic press (Carver Inc., Wabash, IN, USA), and were separated with a 24 µm-thick track-etched polycarbonate membrane with pore diameter of 0.4 or 1 µm (It4ip S.A., Louvain-la-Neuve, Belgium) patterned with an array of holes to provide fluidic access ports to the bottom channel. The microfluidic stack is attached to a modified 384-well COP plate (Aurora Microplates, Whitefish, MT, USA) using a 0.135 mm thick pressure sensitive adhesive consisting of a 25.4 µm polyester carrier film with MA-69 acrylic medical grade adhesive ARcare 90106 (Adhesives Research, Inc., Glen Rock, PA, USA), which had been previously laser-cut in the pattern of the well plate grid and laminated with a hydraulic press at 1.0 MPa for 2 min. Further details on the fabrication process are found elsewhere^28^.

### Integrated micropumps

As described in our previous work^28^, in order to establish recirculating flow in the microchannels, we incorporate a self-priming micropump array into the lid that serves as the fluidic interface with the culture plate via stainless steel tubes. The PREDICT96 pumping system has 192 individual pneumatically-actuated micropumps embedded in the plate lid: two per culture device, one for the culture chamber above the membrane and one for below. However, since the upper chamber is at ALI, only the 96 pumps serving the bottom microchannel are in use during these experiments. Actuation of the pumps transfer media between the microplate wells linked by the bottom channel and establishes a hydrostatic pressure differential, inducing flow through each microchannel.

The pneumatic and fluidic manifolds of the micropump array were constructed with laser-micromachined Ultem polyetherimide and Kapton polyimide films (McMaster-Carr, Elmhurst, Illinois USA) laminated in a heated hydraulic press with phenolic butyral thermosetting adhesive film (R/flex 1000, Rogers Corp., Chandler, Arizona, USA). Fluidic tubing (21G 316L stainless steel hypodermic tubes: New England Small Tube Corp., Litchfield, NH, USA) were glued into the assembly using 353NDPK Hi-Temp Epoxy (Thorlabs, Newton, NJ, USA).

### Trans-epithelial electrical resistance measurement

In order to quantify barrier function, trans-epithelial electrical resistance (TEER) was periodically measured during the ALI differentiation of the PREDICT96-ALI normal human bronchial epithelial (NHBE) tissues. TEER was measured using an Epithelial Volt/Ohm Meter (World Precision Instruments). Blank TEER values were established for each device prior to seeding of NHBEs in each device by adding 100 µL of NHBE proliferation media to each channel. Prior to reading tissue TEER, tissues were incubated at 37 °C for one hour in Hank’s Balanced Salt Solution (HBSS, Sigma) in order to wash away secreted mucus to avoid its interference with TEER reading. After reading TEER values from each device at each time point (generally, 7, 10, 14, 17, 21, 24, and 28 days of culture), TEER in ohms cm^2^ was calculated by subtracting the blank read for each device from the raw reading, and then multiplying this resistance value by the membrane surface area.

### Collection and preparation of human primary bronchial epithelial cells from healthy controls

Participants were recruited via advertisement in the Massachusetts General Hospital (MGH) outpatient clinics and around the Boston metropolitan area. Healthy volunteers between 18 and 50 years old were screened for eligibility with a full medical history. Subjects gave their written informed consent before testing and sample collection. The study was approved by the MGH Institutional Review Board. Volunteers underwent bronchoscopy with conscious sedation. Bronchoalveolar lavage was performed using 4 × 30-ml (mL) aliquots of normal saline. The bronchial mucosa was then sampled using a 4 mm sterile nylon cytology brush. Brush samples were placed in ice-cold RPMI media with 2% human serum and 10 µM ROCK inhibitor (Y-27632, Tocris Bioscience) and cells were gently removed from the brush using a P1000 pipette tip and serially washed with media. The cell suspension was filtered through a 70 µm filter and centrifuged.

### Culture, cryopreservation, and PREDICT96-ALI seeding and differentiation of NHBEs

NHBEs were purchased from Lifeline Cell Technology and Lonza (Table 1). To maintain stocks of each NHBE donor, cells were thawed and plated at 2500 cells/cm^2^ on 804G media-coated tissue culture flasks (804G media made as described elsewhere^31^), cultured in Bronchialife (Lifeline Cell Technology), and passaged using Accutase (Sigma). After two passages, NHBEs were cryopreserved using 65% FBS (Thermo Fisher), 25% Bronchialife (Lifeline Cell Technology), and 10% DMSO (Sigma). Prior to seeding, PREDICT96-ALI plates were sterilized overnight with ethylene oxide gas followed by 1 week of outgassing in a vacuum chamber. PREDICT96-ALI plates were subsequently treated with plasma for 120 s and washed briefly with 70% ethanol, rinsed three times in distilled water, and coated overnight in 804G media. To seed PREDICT96-ALI plates, NHBEs were thawed, counted, re-suspended in complete small airway epithelial cell growth media (SAGM; Lonza), 100 U/mL penicillin–streptomycin (Thermo Fisher), 5 μM ROCKi (Tocris), 1 μM A-83-01 (Tocris), 0.2 μM DMH-1 (Tocris), 0.5 μM CHIR99021 (Tocris) as described elsewhere^32^ (hereafter referred to as SAGM + 4i), and plated at 10,000 cells per device in a 3 μL volume directly onto the membrane. After 48 h of growth in SAGM + 4i media, differentiation was initiated using fresh HBTEC-ALI media (Lifeline Cell Technology) plus 100 U/mL penicillin/streptomycin. After 48 h of submerged differentiation, the ALI was initiated by aspirating media from the apical surface of the tissue in the top chamber, while 60 μL of fresh HBTEC-ALI or custom-ALI media was added to the bottom chamber. Pumping was initiated at 1 μL/min in the bottom microchannel, and the media in the bottom microchannel was changed daily thereafter. Tissues were matured over the course of 3–5 weeks in ALI culture prior to viral infection experiments. At the end of ALI differentiation, the total number of cells per well averages 40,000 (data not shown), and can be determined by enzymatic generation of single cell suspension from mature microtissue and/or by quantification methods of microtissue DNA (Quant-iT PicoGreen dsDNA assay kit, Invitrogen) using sacrificial PREDICT96-ALI devices. To remove accumulated mucus from the apical surface of the maturing tissue, 100 µL of 1 × Hank’s Balanced Salt Solution (HBSS) was added to the apical surface of each tissue and incubated on the tissues for 1 h at 37 °C with rocking, subsequently followed by an additional 5 min wash with 100 µL of 1 × HBSS at room temperature every 7 days (d). Mucus washings were pooled, collected and stored at – 80 °C until processed. Tissue maturity and quality control was scored by a combination of metrics including barrier function, percent ciliated cells, ciliary beat, mucus secretion, and global tissue morphology, and this score was used to determine if individual devices of PREDICT96-ALI airway tissue were suitable for downstream experimentation and viral inoculation. Downstream processing of tissues following viral inoculation was generally conducted at 4–6 weeks of ALI and included tissue apical wash collections, basal media collection, measurement of barrier function, harvest of tissue for RNA extraction, and fixation for immunofluorescence (IF) imaging.

**Table 1:** Summary detailing the primary normal human tracheobronchial epithelial cells (NHBEs) used to grow human airway tissue within the PREDICT96-ALI platform.

Donor no.	Cell type	Vendor/source	Catalogue no.	Batch no.	Age (yo)	Sex	Race
A	NHBE	Lonza	CC-2540S	630564	16	M	C
B	NHBE	Lifeline	FC-0035	05101	29	F	C
C	NHBE	Lifeline	FC-0035	04401	16	M	C
D	NHBE	Massachusetts General Hospital	N/A	DH01	28	F	A

Vendor, catalogue number, batch number (donor lot), age, sex and race of each donor provided.

*M* male, *F* female, *C* Caucasian, *A* Asian (non-Hispanic).

### Immunofluorescence and confocal imaging

To prepare PREDICT96-ALI airway tissues for IF staining, tissues were fixed in situ using 4% paraformaldehyde (Sigma) for 15 min at room temperature rocking and washed three times with 1 × phosphate buffered saline (PBS). Tissues were subsequently permeabilized with 0.3% Triton-100 (Sigma) for 30 min and blocked with 3% normal goat serum (NGS, Thermo Fisher) containing 0.1% Tween-20 (Sigma) for 60 min at room temperature. Primary antibodies for basal cells (CK5, Thermo Fisher), goblet cells (Muc5AC, Thermo), ciliated cells (acetylated-tubulin, Abcam and beta-tubulin, Sigma), cellular proliferation (Ki67, Abcam), influenza A nucleoprotein (IAV-NP, Abcam), and ACE2 (R&D Systems) were diluted 1:50 (ACE2 only) or 1:100 in 3% NGS including 0.1% Tween-20 and incubated overnight at 4 °C with rocking. Devices were washed twice with PBS followed by one rinse with 3% NGS plus 0.1% Tween-20 for 5 min each with rocking and secondary antibodies Alexa Fluor 488 goat anti-mouse IgG (Thermo Fisher) and Alexa Fluor 555 goat anti-rabbit IgG (Thermo Fisher) were diluted 1:300 and incubated for 18 h at 4 °C with rocking. Secondary antibodies were removed by washing three times with PBS containing 0.1% Tween-20, and tissues were incubated with rocking at room temperature for 30 min with Hoechst 33342 nuclear stain (Thermo Fisher, 10 mg/mL stock) at a 1:800 dilution and Phalloidin-iFluor 647 Reagent (Abcam) at 1:1000. After 30 min, the PREDICT96-ALI tissues were washed at least three times for 5 min with PBS prior to imaging. Stained PREDICT96-ALI devices were imaged using a Zeiss LSM700 laser scanning confocal microscope and Zen Black software. Tile scans and z-stacks of the tissue at 40 × magnification were acquired. Quantification of mean fluorescence intensity was done using ImageJ, version 1.5.2e (Wayne Rashband, National Institutes of Health, USA).

### Viral stocks, propagation, and titer

Influenza A viral strains A/California/04/09 H1N1 (ATCC), and A/Hong Kong/8/68 H3N2 (Charles River) were used for experiments. IAV strains were propagated in MDCK cells (ATCC), harvested in Dulbecco's Modified Eagle Medium (DMEM, Thermo Fisher) containing 7.5% bovine serum album (BSA, Invitrogen), 100 U/mL penicillin–streptomycin (Invitrogen), and 0.1 µg/mL *N*-tosyl-l-phenylalanine chloromethyl ketone-trypsin (TPCK-trypsin, Sigma) and titered by viral plaque assay using MDCK cells as previously reported^33^. Viral infection experiments in PREDICT96-ALI tissue used A/California/04/09 H1N1 at passage 3, and A/Hong Kong/8/68 H3N2 at passage 2.

Human coronavirus strain (HCoV) NL63 was obtained through BEI Resources, NIAID, NIH, Human Coronavirus NL63, NR-470) and propagated two times in Lilly Laboratories Cell-Monkey Kidney 2 (LLC-MK2) using a method adapted from Herzog^34^. In brief, HCoV-NL63 was grown using LLC-MK2 cells in a limiting dilution series. Recovered virus was harvested from the last well of the dilution series showing diffuse cytopathic effect (CPE) at 4–5 d post-infection (p.i.). Subsequently, confluent LLC-MK2 monolayers were inoculated in 25 cm^2^ flasks with a 1:10 dilution of viral supernatant from the prior viral passage. The flasks were incubated at 34 °C, 5% CO_2_, and harvested on day 4 when diffuse CPE was observed. To harvest the virus, flasks were frozen at − 80 °C and thawed at 37 °C twice, and cells and supernatant were pooled and centrifuged at 4 °C for 10 min at 3000 rpm. Cleared supernatant was aliquoted and stored at − 80 °C, and is referred to henceforth as passage 2 virus. HCoV-NL63 was harvested in EMEM (ATCC) containing 0.1% BSA (Sigma) and 100 U/mL penicillin–streptomycin (Thermo Fisher). Viral infection experiments with PREDICT96-ALI tissue used HCoV-NL63 at passage 2.

Viral plaque assay using LLC-MK2 cells were used to titer propagated HCoV-NL63. A tenfold serial dilution was performed using the propagated virus, followed by 1 h of viral binding (100 µL of appropriate viral dilution per well of 12-well plate) with rocking at 34 °C on confluent LLC-MK2 cells. After unbound virus was removed with one wash of phosphate-buffered saline (PBS), the cells were overlaid with agar (0.3%) in EMEM supplemented with 0.1% BSA and 100 U/mL penicillin–streptomycin. After the agar solidified at room temperature, the plates were incubated for 6 d at 34 °C until diffuse CPE was observed. The gel overlay was removed and the wells washed once with PBS. Cells were fixed using 4% paraformaldehyde (Sigma) overnight at 4 °C and stained with 0.1% crystal violet (Sigma) in 4% ethanol (Sigma) for 30 min at room temperature. Stained cells were washed in PBS and imaged, and plaque counts were determined using images analyzed using a customized MATLAB script to determine the plaque forming units (PFU) per mL.

### Oseltamivir dosing

Oseltamivir carboxylate (F. Hoffmann-La Roche Ltd., Basel, Switzerland) was used in anti-viral screens performed on PREDICT96-ALI tissue. Oseltamivir in distilled water was diluted to 0 (vehicle control), 0.01, 0.1, 1, or 10 µM in complete HBTEC-ALI or custom-ALI media and applied to the bottom channel of PREDICT-ALI airway tissues subject to anti-viral evaluation following the standard apical wash of the tissue and 2 h prior to IAV-inoculation. Upon and after IAV-inoculation of the apical side of the PREDICT96-ALI tissue, 0 (vehicle control), 0.01, 0.1, 1, or 10 µM oseltamivir was maintained in the basal media for the duration of the study. Basal media changes including oseltamivir occurred every 24 h.

### Camostat mesylate treatment methods

Camostat mesylate (R&D Systems, BioTechne Corp., Minneapolis, MN) was used on PREDICT96-ALI tissue infected with coronavirus strain HCoV-NL63. Camostat mesylate in DMSO was diluted to 0 (vehicle control) or 20 µM in complete HBTEC-ALI or custom-ALI media and applied to the bottom channel of PREDICT-ALI airway tissues subject to anti-viral evaluation following the standard apical wash of the tissue, and 2 h prior to HCoV-NL63 inoculation. Upon and after HCoV-NL63 inoculation of the apical side of the PREDICT96-ALI tissue, 0 (vehicle control) or 20 µM camostat mesylate was maintained in the basal media for the duration of the study. Basal media changes including camostat mesylate occurred every 24 h.

### Infection of PREDICT96-ALI tissues with viral strains

To prepare PREDICT96-ALI tissues for influenza and coronavirus infection, a mucus wash with 1 × HBSS was performed as described above. Viral strains were thawed on ice and individually diluted in viral infection media to reach the relevant MOI. Influenza virus infection media was composed of a single IAV strain diluted to the appropriate MOI in HBSS including 1 µg/mL TPCK-trypsin (Sigma) for A/California/04/09 and A/Hong Kong/8/68. Coronavirus infection media was composed of HCoV-NL63 diluted to the appropriate MOI in HBSS. When the mucus wash was completed, viral inoculum was added to the apical side of the tissues. Each experiment included both an untreated control in which ALI was maintained and an untreated control that was submerged in HBTEC-ALI or custom-ALI media for comparison to the viral inoculum-treated groups. The time of incubation varied by viral strain, with HCoV-NL63 incubated on the PREDICT96-ALI tissues for 6 h with rocking and A/California/04/09 and A/Hong Kong/8/68 each individually incubated for 1 h with rocking. Following incubation, both the top chamber and the bottom channel were aspirated, the apical surface of the tissue washed three times with 1 × HBSS with the final wash collected for reference, and the top chamber was completely aspirated to resume ALI culture. A volume of 60 μL of HBTEC-ALI or custom-ALI media was added to the bottom channel to resume normal culture conditions, with 1 µg/mL TPCK-trypsin maintained in the media of devices that had been inoculated with A/California/04/09 and A/Hong Kong/8/68. At 24 or 48 h intervals p.i., an apical wash using 1 × HBSS was performed at 34–37 °C for 1.5 h with rocking to collect both mucus and virus. Specifically, 100 µL of 1 × HBSS was added to the apical surface of each tissue and incubated on the tissues for 45 min at 34–37 °C with rocking, subsequently followed by an additional 50 µL wash with 1 × HBSS for 45 min at 34–37 °C with rocking and a final 50 µL wash with 1 × HBSS for 5 min at room temperature. Apical washings were pooled, collected and stored at − 80 °C until processed. Basal media was collected at time points p.i. that correspond to mucus and virus wash collections, and stored at − 80 °C. Following apical wash and basal media collections, 60 μL of HBTEC-ALI or custom-ALI media (including 1 µg/mL TPCK-trypsin for devices that had been inoculated with either A/California/04/09 or A/Hong Kong/8/68) was added to the bottom channel, and complete aspiration of the top chamber to resume ALI culture was performed to re-establish normal culture conditions.

### RNA extraction and RT-qPCR

Supernatant viral RNA was collected from the apical side of PREDICT96-ALI tissues and isolated using a QIAamp Viral RNA Mini Kit (Qiagen) following the manufacturer’s specifications. Supernatant volumes of 100 μL were brought to 140 μL using HBSS. In order to collect bulk tissue RNA from devices, RLT buffer (Qiagen) with 0.01% v/v 2-mercaptoethanol (Sigma) was added to both the top chamber and bottom channel to disrupt the differentiated tissue. Tissue RNA was extracted from PREDICT96-ALI devices using an RNeasy Micro Kit (Qiagen) per the manufacturer’s instructions. One-step quantitative reverse transcription polymerase chain reaction (RT-qPCR) was then performed on extracted RNA samples using a QuantiTect Probe RT-PCR kit (Qiagen) following the standard protocol for a QuantStudio 7 Flex RT-PCR system. Briefly, 7.8 μL of the extracted supernatant viral RNA was used in a 20 μL reaction volume and 3.8 μL tissue RNA was used in a 20 μL reaction volume, and samples were run in duplicate. The reaction was run in an Applied Biosystems QuantStudio 7 Flex System (Thermo Scientific) using the following condition: 50 °C for 20 min, 95 °C for 5 min, 40 cycles of 95 °C for 15 s and 60 °C for 45 s. TaqMan primers and probe targeting IAV-M were ordered from Thermo Scientific with the following sequences: FLUAM-7-F: CTTCTAACCGAGGTCGAAACGTA, FLUAM-161-R: GGTGACAGGATTGGTCTTGTCTTTA, FLUAM-49-P6: TCAGGCCCCCTCAAAGCCGAG, and TaqMan primers and probe targeting HCoV-NL63 (Assay ID: Vi06439673_s1) were ordered from Thermo Scientific. TaqMan Gene Expression Assays (Thermo Scientific) were used to target the following proteins in lung tissue: ACE2 (Hs01085333_m1), TMPRSS2 (Hs01122322_m1), TUBB6 (Hs00603164_m1), and Muc5AC (Hs01365616_m1). Absolute quantification (copies/mL) of supernatant viral RNA was calculated using a standard curve generated from serial dilutions of A/PR/8/34 viral RNA (Charles River Laboratories) or linearized HCoV-NL63 viral RNA (ATCC). Comparative cycle threshold (Ct) values were determined using the method described by Schmittgen and Livak^35^ using normalization to the housekeeping gene GAPDH.

### Statistical analysis

Data are presented as mean ± standard deviation and were analyzed using GraphPad Prism version 8.3.1 for Windows (GraphPad Software). Statistical significance was determined using one- or two-way analysis of variance (ANOVA) with Tukey’s or Sidak’s post hoc test for multiple comparisons, where appropriate. A p value lower than 0.05 was considered statistically significant, and is indicated in figures as follows: *p ≤ 0.05, **p ≤ 0.01, ***p ≤ 0.001, and ****p ≤ 0.0001.

### Ethics statement

All research involving human subjects was approved by the MGH Institutional Review Board, and adheres to all applicable institutional and sponsor ethical guidelines. All methods are in adherence with applicable safety and laboratory practice guidelines.

## Results

### Creation of a high-throughput ALI platform for dynamic culture of human airway tissue: PREDICT96-ALI

The adaptation of the PREDICT96 platform^28^ for the ALI model allows for inoculation of cultured tissues in each channel apically or basally, sampling of the basal media at regular intervals, and examination of the tissues at the completion of each study, as shown in Fig. 1A. The schematic (left) illustrates the 384 well standard structure upon which each of the individual airway models is situated. At the right, the detailed view shows access to the top chamber (top two wells of the 2 × 2 array) and the bottom channel (accessed by an inlet and outlet from the bottom two elements of the 2 × 2 array, respectively). An exploded-view schematic (Fig. 1B) of this configuration depicts the assembly of the material layers required to establish the microfluidic structure of the PREDICT96-ALI platform. Photographs in Fig. 1C depict the PREDICT96-ALI platform from an upright view (left), inverted view (right) and overhead view (bottom), with a higher magnification detail of an individual airway model.

### PREDICT96-ALI recapitulates healthy human airway structure and composition

**Figure 2:**
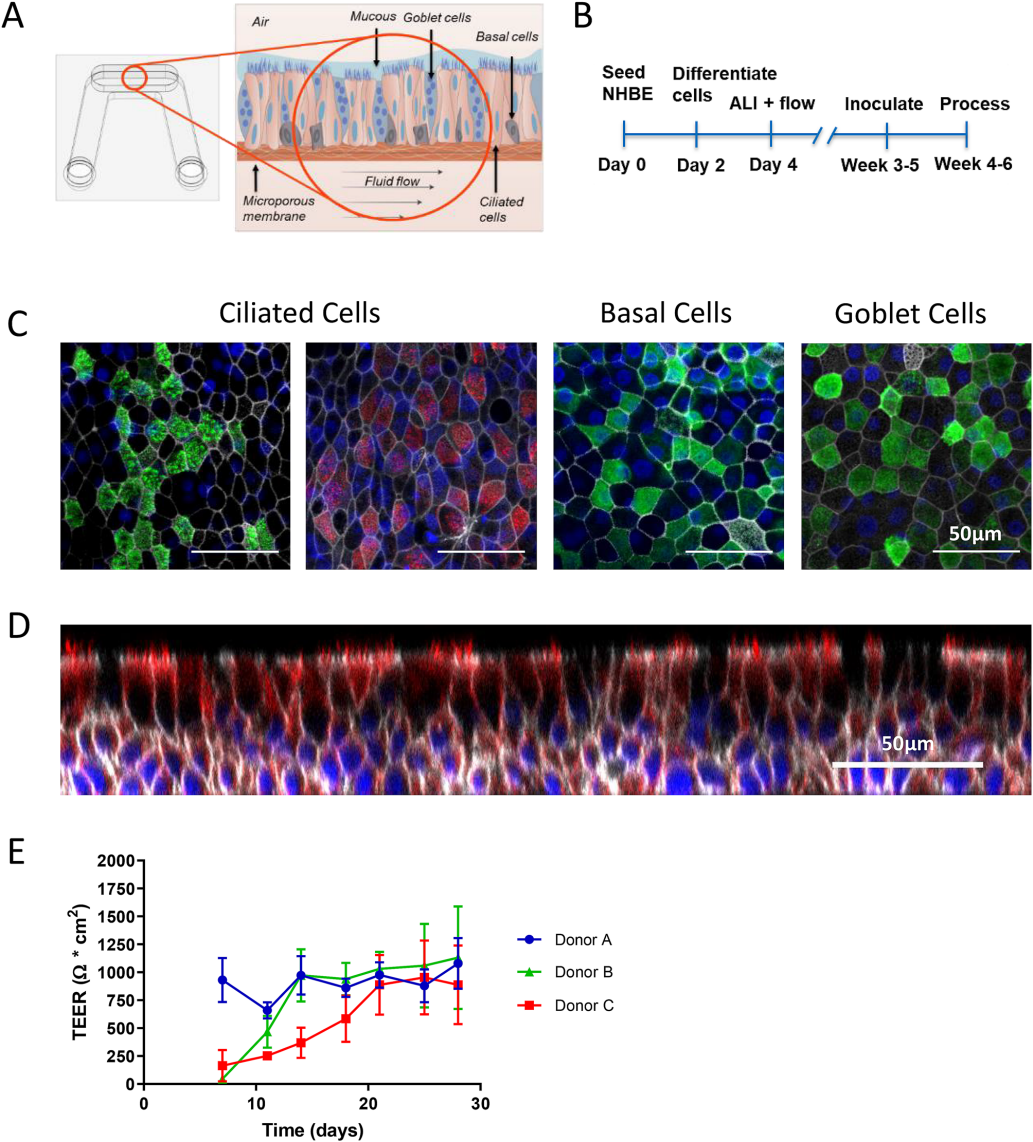
Healthy PREDICT96-ALI airway model. (**A**) Schematic illustrating the configuration of the biomimetic airway model within the PREDICT96-ALI platform with associated microenvironmental features including differentiated cell populations (ciliated, goblet, basal and club cells) of the mature tissue, apical mucus and periciliary fluid present on mature human airway, and basolateral fluid flow to recirculate nutrients, remove waste products, and oxygenate the media. (**B**) Timeline detailing proliferation, differentiation, inoculation and processing (sample collection, fixation, imaging, etc.) of the PREDICT96-ALI airway tissue over 4–6 weeks. (**C**) Stains of ciliated (acetylated-tubulin, green; β-tubulin, red), basal (CK5, green), and goblet (Muc5ac, green) cells counterstained for nucleic acids (DAPI, blue) and actin (phalloidin, grey) at ×40 magnification shown, with a 50 µm scale bar. Donor B is featured as representative. (**D**) High-resolution confocal microscopy ×40 z-stack orthogonal image of the pseudostratified epithelium with approximately 4 cell layers and 50 µm thickness established after culture at 28 days at an ALI. Donor B is featured as representative. (**E**) Transepithelial electrical resistance (TEER) plotted over time for three human primary epithelial cell donor populations, showing that the same ultimate level of barrier function is reached, although the kinetics of barrier resistance are different.

First, the PREDICT96-ALI system was used to establish the healthy baseline airway model, prepared as described in the Methods section, and morphology and cell populations were evaluated using high resolution confocal microscopy and immunohistochemistry. An illustration depicts the development of a healthy airway model within PREDICT96-ALI (Fig. 2A). The experimental timeline and setup for the system is outlined in Fig. 2B. Fluorescent staining of ciliated cells, goblet cells, and basal cells (Fig. 2C), as well as a cross-section of the pseudostratified epithelial layer (Fig. 2D) are shown for a typical airway tissue within a single device of PREDICT96-ALI. Our observations for airway tissues in the PREDICT96-ALI platform regarding the relative populations of ciliated cells, basal cells, and goblet cells are consistent with our previously reported observations in earlier generations of our airway models as well as with in vivo observations^26^. We also evaluated TransEpithelial Electrical Resistance (TEER) for the airway tissues over time during establishment of the ALI, observing varying kinetics of barrier resistance across different human donor populations but ultimately the same final level of TEER for a given media formulation and protocol (Fig. 2E). Note that the Lonza donor (donor A) exhibited a rapid increase in TEER, while the two Lifeline donors (donors B and C) showed a slow rise to approximately the same final value; the reasons for these differences are unclear, but the ability of this platform to assess these donor-to-donor differences within a single plate is potentially quite useful. We have also observed that the behavior of TEER is strongly dependent upon media formulation, which can shift the baseline TEER behavior between different conditions.

**Figure 3:**
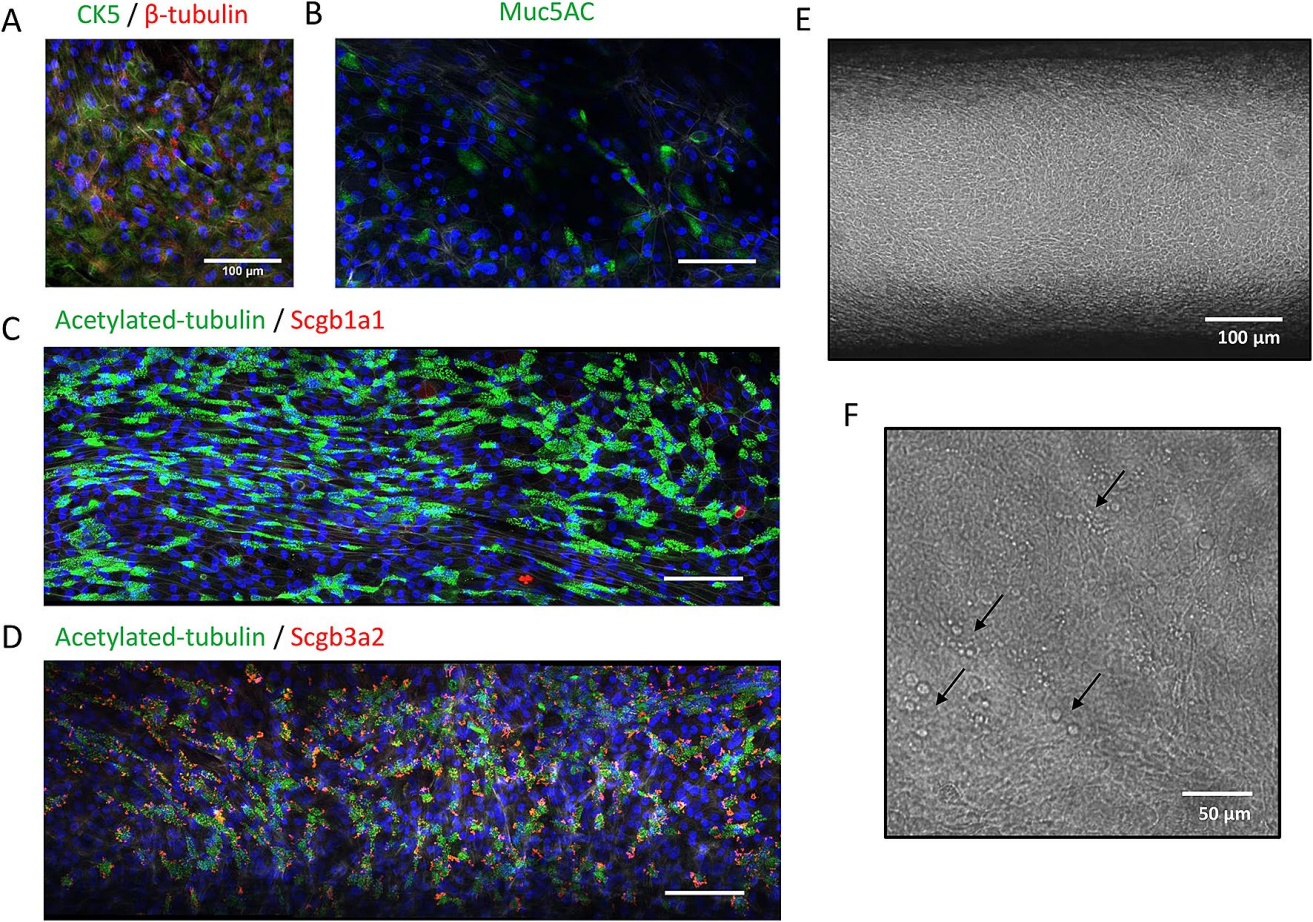
Healthy PREDICT96-ALI airway model with freshly harvested epithelial cells from research bronchoscopies. Panels illustrating establishment of mature tissue with pseudostratified morphology, approximately 30–50 µm, thick from freshly harvested airway epithelial cells obtained from living research bronchoscopy donor D. (**A–D**) ×40 images showing basal cell populations (CK5, green), goblet cells (Muc5AC, green), ciliated cells (acetylated tubulin, green; β-tubulin, red), and club cells (Scgb1a1, red; Scgb3a2, red) counterstained with nucleic acid (DAPI, blue) and actin (phalloidin, grey) with scale bar 100 µm, (**E**) ×10 phase contrast image of mature tissue at 28 days at an ALI with scale bar 100 µm, and (**F**) ×40 phase contrast image of mucus droplets (black arrows) on surface of mature tissue at 28 days at an ALI with scale bar 50 µm. Cells are characterized by robust ciliary beat (see supplementary video) and mucociliary flow.

We explored the formation of a pseudostratified epithelium at an ALI using freshly harvested human bronchial epithelial cells from living research bronchoscopies, labeled DH01, observing robust establishment of airway tissues over periods of 5 weeks or longer in culture in the PREDICT96-ALI plates. Panels illustrating the establishment of mature tissue with a pseudostratified morphology, approximately 30–50 μm thick, are provided in Fig. 3A–D. Here we show ×40 images of IF staining of cell populations as described above, including basal cell populations (CK5), goblet cells (Muc5AC), ciliated cells (acetylated tubulin, beta-tubulin), and club cells (scgb1a1, scgb3a2), all with scale bar 100 μm. We observed that club cells are clearly visible in IF staining with this donor; their presence varies between donors and assays, and there are reports suggesting a key role club cells play in the immunopathology of IAV^36^. Also shown is the morphology of mature tissue cultured in the PREDICT96-ALI, shown *en face* at 10 × magnification (Fig. 3E), and the presence of mucus droplets formed on the surface of the mature tissue at ×40 (Fig. 3F); we also observe robust ciliary beating (see supplementary video 1) and mucociliary flow.

### PREDICT96-ALI airway model supports infection with IAV

**Figure 4:**
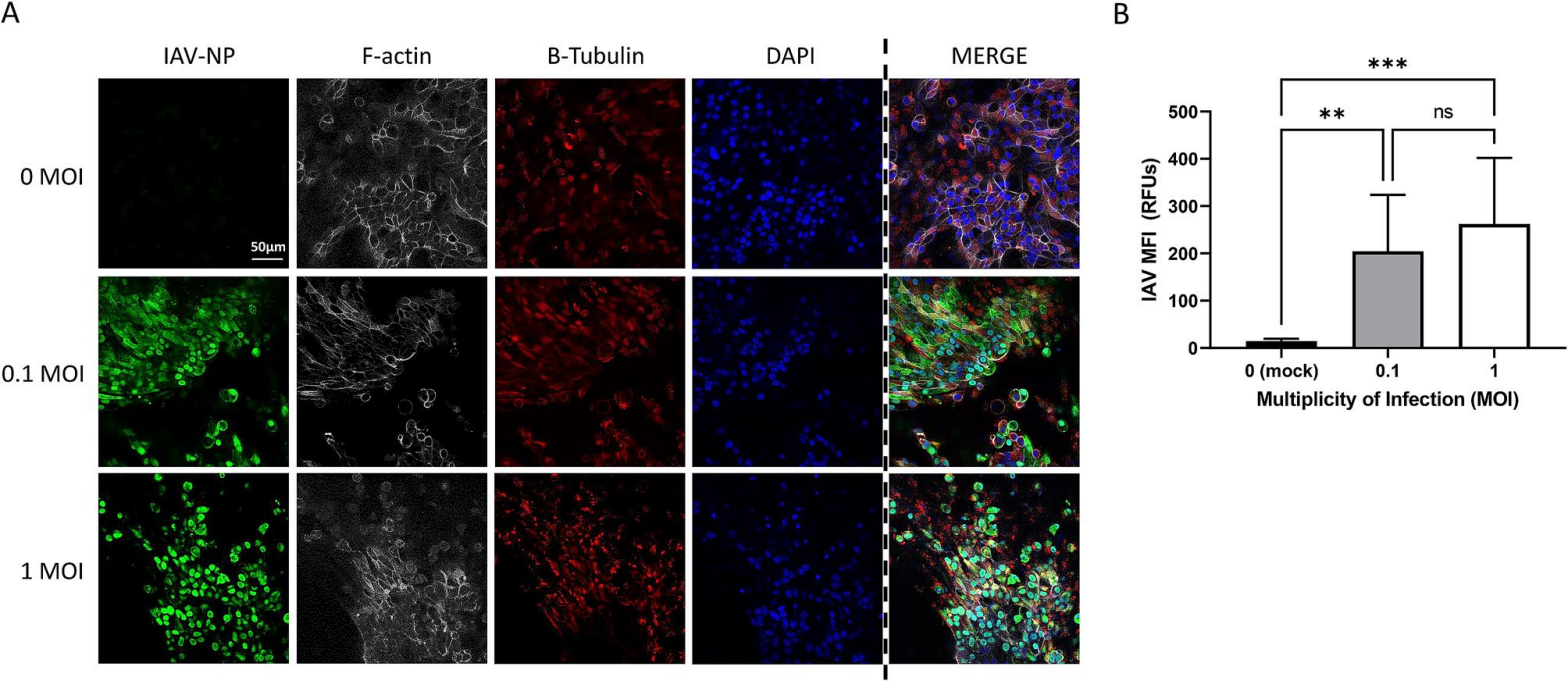
Immunofluorescence staining and quantification of IAV-infected PREDICT96-ALI airway tissue. (**A**) Staining of the nucleoprotein (NP, green) for IAV, actin (phalloidin, grey), β-tubulin (red) and nucleic acids (DAPI, blue), including merged panels for all four stains, within PREDICT96-ALI airway tissue developed using freshly harvested epithelial cells from donor D at 48 h p.i., after 5 weeks ALI culture and following inoculation with A/California/04/09 H1N1 (MOI 0 [mock control], 0.1 or 1). Strong expression of IAV-NP is seen at 48 h p.i. at both MOI 0.1 and 1; noted here is that this is one field of view (FOV), and that there is more cytopathic effect at MOI = 1. Images captured are ×40, z-stacks, slice 25 of 48. Scale bar 50 µm. (**B**) Quantification of the mean fluorescence intensity (MFI) of the IAV-NP stain (green) or Alexa Fluor 488 signal, indicating significant increase in IAV replication at both 0.1 and 1 MOIs. N = 2 independent experiments with data presented from donor D as representative. Per experiment, N = 3 tissue replicates per donor, time-point and condition (MOI, anti-viral dose). One FOV was analyzed at N = 7 slices (at 5 slice intervals) for each condition. Statistical significance: **p ≤ 0.01 and ***p ≤ 0.001.

Next, we examined the effect of inoculation of airway ALI cultures with various strains of IAV, including A/California/04/09 H1N1 and A/Hong Kong/8/68 H3N2. In Fig. 4, IF staining of ALI culture devices seeded with living donor D cells and inoculated with various MOIs of A/California/04/09 H1N1 are shown, demonstrating greater abundance of IAV nucleoprotein (NP) as the MOI increases. The 40 × images at the apical plane show IAV-NP staining across the range of 0 (mock control)–1 MOI, with levels increasing substantially relative to mock control. We also saw an observeable but non-significant increase in IAV-NP staining between 0.1 and 1 MOIs. Donors exhibited varied cytopathic response to increasing MOIs, with the living research bronchoscopy donor D cells showing the strongest cytopathic response at higher MOI, and the commercial vendor-derived donors A, B, and C displaying the greatest resilience to MOIs ≥ 1 (data not shown). The earlier observations regarding the presence of club cells in certain donor lots may be relevant to the cellular tropism of IAV strains such as A/California/04/09 H1N1 and A/Hong Kong/8/68 H3N2, potentially impacting donor-specific cytopathic response and susceptibility to IAV.

**Figure 5:**
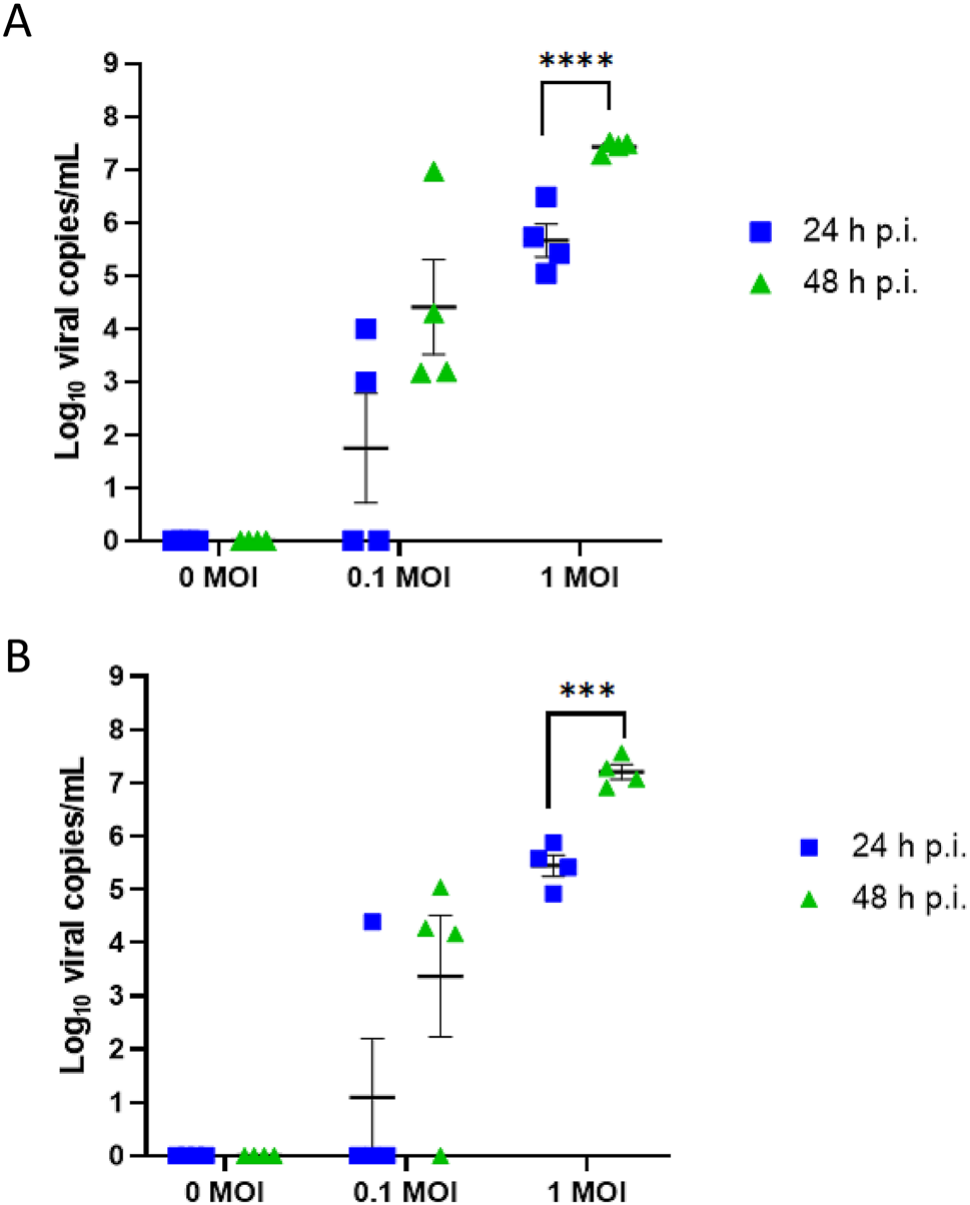
Infection kinetics of IAV-inoculated PREDICT96-ALI airway tissue. RT-qPCR analyses for viral copies in the apical wash of PREDICT96-ALI airway tissue at 24 and 48 h p.i. (**A**) PREDICT96-ALI airway tissue inoculated with A/California/04/09 H1N1 and monitored for an increase in viral load at 24 h intervals with a statistically significant increase in viral copies at 48 h p.i. compared to 24 h p.i. for the MOI 1 condition. (**B**) PREDICT96-ALI airway tissue inoculated with A/Hong Kong/8/68 H3N2 and monitored for an increase in viral load at 24 h intervals with a statistically significant increase in viral copies at 48 h p.i. compared to 24 h p.i. for the MOI 1 condition. Statistical significance: ***p ≤ 0.001 and ****p ≤ 0.0001. N = 2 independent experiments with data presented from one representative experiment. Per experiment, N = 3–4 tissue replicates per donor, time-point and condition. Donor B showcased as representative. Replicates that did not meet the minimum signal intensity after 45 PCR cycles are displayed along the x-axis.

Measurements of supernatant viral RNA were obtained using RT-qPCR, and are provided in the two panels in Fig. 5. Viral copy numbers after inoculation at MOIs of 0.1 and 1 (including mock control, MOI 0) were measured at 24 and 48 h p.i. for the A/California/04/09 H1N1 strain of IAV (Fig. 5A). In Fig. 5B, companion results for A/Hong Kong/8/68 H3N2 are shown. For the H1N1 and H3N2 strains, viral copies per mL tend to increase with MOI, and continue to rise between 24 and 48 h p.i. The RT-qPCR measurements are a useful readout of infection but are prone to error due to a positive signal arising from dead virus, and therefore the IF data from the previous section, as well as plaque assays planned for future studies, represent key additional metrics.

### IAV replication is reduced by oseltamivir in PREDICT96-ALI airway model

**Figure 6:**
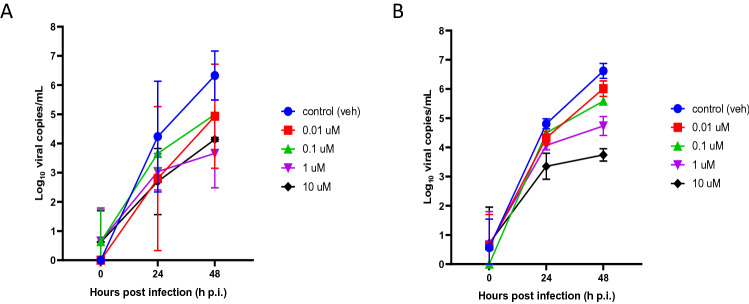
Infection Kinetics of IAV-inoculated PREDICT96-ALI Airway Tissue in Response to Oseltamivir. RT-qPCR analyses for viral copies of the apical wash of PREDICT96-ALI airway tissue at 24 and 48 h p.i. in absence (blue, vehicle control) or presence (red = 0.01 µM, green = 0.1 µM, violet = 1 µM, black = 10 µM) of oseltamivir (Tamiflu) for two donors labeled donor B (**A**) and donor C (**B**). PREDICT96-ALI airway tissue inoculated with A/Hong Kong/8/68 H3N2 at MOI = 0.1, and monitored for viral load over 24 h intervals up to 48 h p.i. with a marked decrease in viral copies in response to oseltamivir dose at both time points. N = 2 independent experiments with data presented from one representative experiment. Per experiment, N = 3–4 tissue replicates per donor, time-point and condition. Replicates that did not meet the minimum signal intensity after 45 PCR cycles are displayed along the x-axis.

To determine if the PREDICT96-ALI airway model can be used to evaluate the efficacy of potential antiviral therapeutics, we investigated the effect of the antiviral agent oseltamivir—the most commonly used clinical anti-influenza therapy—for its ability to reduce viral load in IAV-inoculated PREDICT96-ALI airway tissue. The active form of oseltamivir, oseltamivir carboxylate, was introduced 2 h before viral inoculation and maintained in the bottom channel of the PREDICT96-ALI airway tissues during the course of infection with A/HongKong/8/68 H3N2 virus at various MOIs. Oseltamivir (> 0.1 μM) significantly reduced influenza replication up to 48 h p.i. (Fig. 6, Supplemental Fig. 1) relative to control tissue devices for the A/Hong Kong/8/68 H3N2 strain at MOI 0.1 and prevented virus-induced disruption to barrier function and epithelial tight junction formation (data not shown). An MOI of 0.1 was selected for the oseltamivir dosing study to remain well below the threshold for significant cytopathic effects at MOI 1, an observation often seen at higher MOI in IAV inoculation studies. Although oseltamivir was able to reduce IAV replication in the donors investigated, donor-specific behaviors were observed in response to viral infection. Fabrication-related parameters associated with the platform may represent an additional source of variability beyond potential biological variability from donor-to-donor. Measurements for viral copies in the PREDICT96-ALI airway model reflect those achieved in clinical settings among patients exposed to influenza and treated with oseltamivir^37–40^, suggesting that the PREDICT96-ALI airway model can serve as a preclinical tool to evaluate potential therapies for combating respiratory infections of the human airway.

### ACE2 and TMPRSS2 expression is confirmed in PREDICT96-ALI airway model

**Figure 7:**
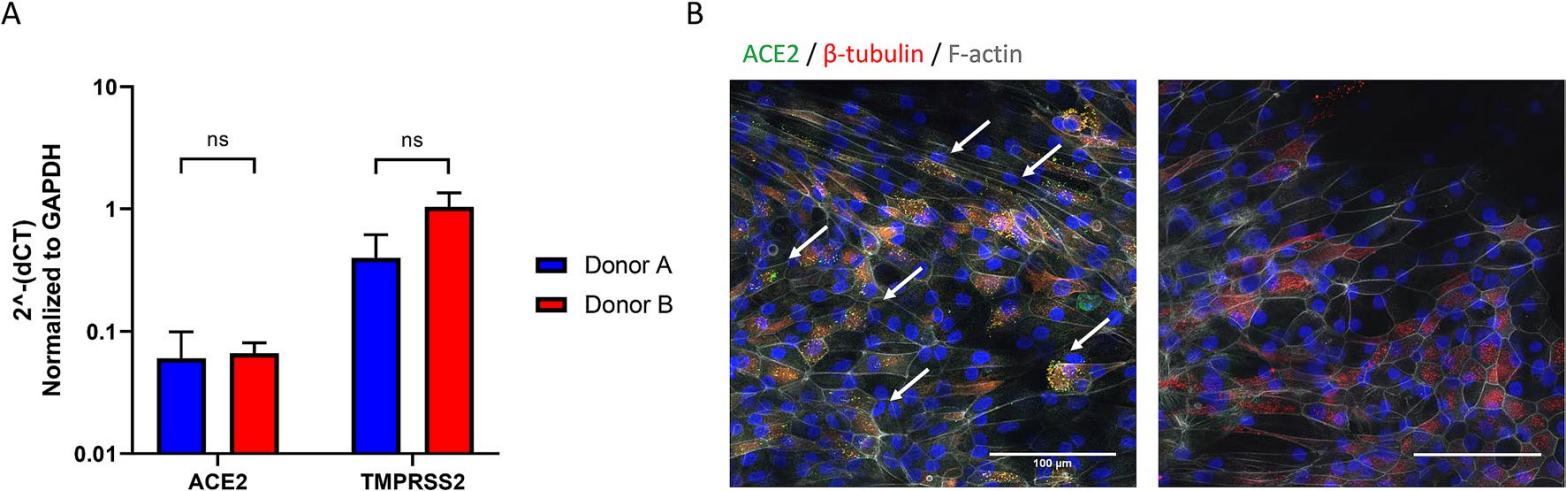
Characterization of ACE2 and TMPRSS2 expression in PREDICT96-ALI airway tissues. (**A**) Transcripts of ACE2 and TMPRSS2, both critical for establishing HCoV-NL63 and SARS-CoV-2 infection, were detected by RT-qPCR from PREDICT96-ALI airway tissue. (**B**) ACE2 protein expression was detected (left panel) on the apical surface of the tissue by in-situ IF in PREDICT96-ALI airway tissues (donor D). Representative ×40, confocal image (mean fluorescence intensity) showing ACE2 (green, red arrows) counterstained with nucleic acid (DAPI, blue) and actin (phalloidin, grey) compared to secondary-only control (right panel) with scale bar 100 µm. PREDICT96-ALI airway tissue from donors A and B were matured to 4 weeks ALI and shown to have a non-significant (ns) difference in ACE2 and TMPRSS2 gene transcript levels and ACE2 protein levels (data not shown) when compared to one another. Comparative CT values were determined using the method described by Schmittgen and Livak^35^ and used to determine the relative quantification of gene expression using GAPDH as a reference gene. N = 3–4 tissue replicates per donor for both RT-q-PCR and IF.

In preparation for testing the viral respiratory infection model with coronaviruses, we probed for transcript and protein expression of cell surface proteins ACE2 and TMPRSS2, known to play central roles in facilitating viral infection by SARS-CoV-2^41^. Both HCoV-NL63 and SARS-CoV-2 gain entry into host cells via the ACE2 receptor, while TMPRSS2 facilitates SARS-CoV-2 infection via cleavage mechanisms, and thus its presence is an important aspect of the model. Data for ACE2 and TMPRSS2 transcripts for cell populations derived from two different donors are plotted on a semi-logarithmic scale in Fig. 7A, and data for ACE2 protein expression on the apical surface of the tissue derived from donor B in Fig. 7B. Consistent with other reports of ACE2 expression in human donor airway tissue, ACE2 is localized to the apical surface of the microtissues and displays punctate appearance, indicating possible association with the motile cilia of multiciliated airway epithelial cells^42^. Data from each donor evaluated show consistent transcript expression for ACE2 and TMPRSS2 and protein expression for ACE2 between donors (data not shown). The consistency between these key cellular factors among different donors is encouraging, as it suggests that the platform is capable of supporting proliferation and maturation of tissues derived from numerous donors. Thus, evaluation of many donors can be accommodated in the PREDICT96-ALI system both as a healthy model and in the presence of pathogenic challenge. Evaluation of protein expression by IF or western blot of TMPRSS2 and other cell surface proteins known to play key roles in facilitating coronavirus infection would further inform donor assessment and viral entry mechanisms.

### PREDICT96-ALI airway model supports infection with HCoV-NL63

**Figure 8:**
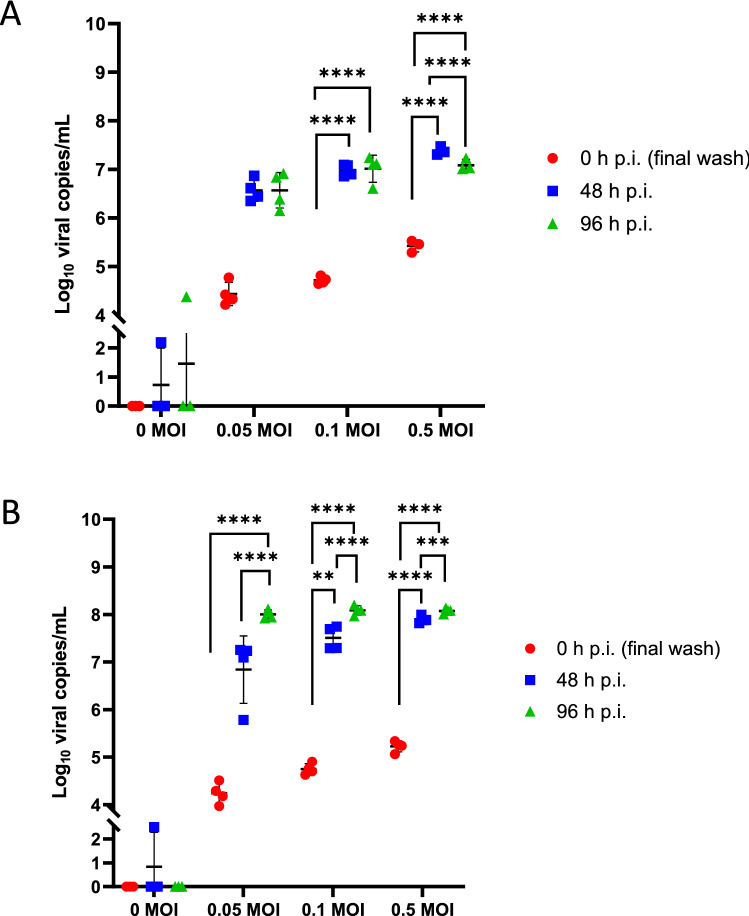
Infection kinetics of HCoV-NL63-inoculated PREDICT96-ALI airway tissue. RT-qPCR analyses for viral copies in the apical wash of PREDICT96-ALI airway tissue at 0, 48 and 96 h p.i. for two human donors showing unique infection kinetic profiles. (**A**) PREDICT96-ALI donor A airway tissue inoculated with HCoV-NL63 and monitored for an increase in viral load at 48 h intervals with a statistically significant increase in viral copies at 48 and 96 h p.i compared to 0 h p.i. for the MOI 0.1 and 0.5 conditions. Statistical significance: ****p ≤ 0.0001. (**B**) PREDICT96-ALI donor B airway tissue inoculated with HCoV-NL63 and monitored for an increase in viral load at 48 h intervals with a statistically significant increase in viral copies at 96 h p.i compared to 48 and 0 h p.i. for MOI 0.05, 0.1, and 0.5, as well as a statistically significant increase in viral copies at 48 h p.i. compared to 0 h p.i. for MOI 0.1 and 0.5 conditions. Transient, non-productive viral infection detected at 48 h p.i. in mock condition (0 MOI), is likely the result of technical handling by the experimental operator. Statistical significance: **p ≤ 0.01, ***p ≤ 0.001, and ****p ≤ 0.0001. N = 2 independent experiments with data presented from one representative experiment. Per experiment, N = 3–4 tissue replicates per donor, time-point and condition. Replicates that did not meet the minimum signal intensity after 45 PCR cycles are displayed along the x-axis.

We have also investigated viral infection with HCoV-NL63 in the PREDICT96-ALI culture system with primary NHBEs, as shown in Fig. 8A,B for two different donor cell populations. In each case, viral copies in the supernatant were measured with RT-qPCR over periods extending to 96 h p.i. and across a range of MOIs, include a mock condition (0 MOI). We observed a clear increase in viral load over these time periods for both donors, indicating viral propagation in the airway tissues. Viral copies rose sharply from 0 to 48 h for each MOI tested, while from 48 to 96 h the rate of viral propagation decreased and differed between the two donors. We believe that the positive detection of viral particles in some of the PREDICT96-ALI devices exposed to mock condition (0 MOI) was likely associated with user-introduced technical error, and the transience of the positive detection was likely the result of non-productive infection, as indicated by the elimination of the positive signal at later time points in the experimental time-course. These observations may also be associated with thresholding error, since the increases by approximately 1 log around the 2–6 day p.i. time points are small enough that the data are within potential measurement noise.

### Viral infections kinetics are modeled for HCoV-NL63 and HCoV-OC43

**Figure 9:**
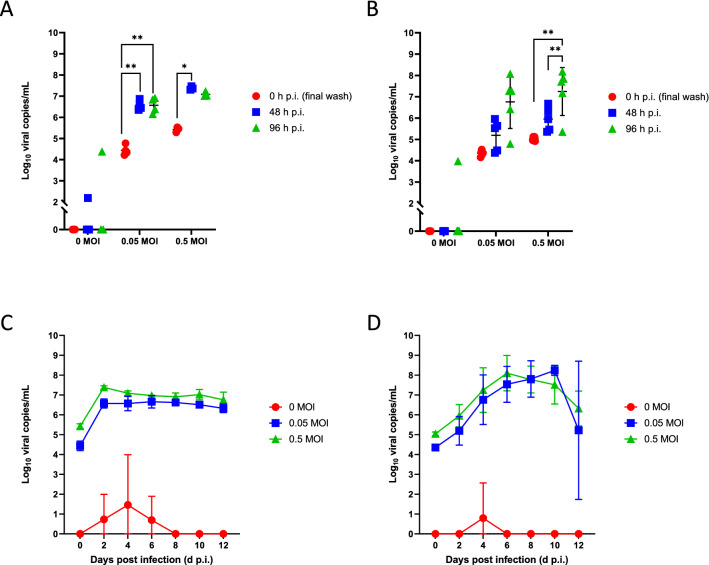
Infection of coronavirus strains in PREDICT96-ALI model. RT-qPCR analyses for viral copies of HCoV-NL63 (**A**,**C**) and HCoV-OC43 (**B**,**D**) in the apical wash of PREDICT96-ALI airway tissue model from a single donor, measured over 12 days across three different MOIs (0, 0.05, 0.5). (**A**) PREDICT96-ALI airway tissue inoculated with HCoV-NL63 and monitored for an increase in viral load at 48 h intervals with a statistically significant increase in viral copies at 48 and 96 h p.i. compared to 0 h p.i. for the MOI 0.05 condition, as well as a statistically significant increase at 48 h p.i. compared to 0 h p.i. for the MOI 0.5 condition. (**B**) PREDICT96-ALI airway tissue inoculated with HCoV-OC43 and monitored for an increase in viral load at 48 h intervals with a statistically significant increase in viral copies at 48 and 96 h p.i. compared to 0 h p.i. for the MOI 0.5 condition. Statistical significance: *p ≤ 0.05 and **p ≤ 0.01. N = 4–5 tissue replicates per donor, time-point and condition. Replicates that did not meet the minimum signal intensity after 45 PCR cycles are displayed along the x-axis. Viral copy number versus day post-infection shown for HCoV-NL63 (**C**) and HCoV-OC43 (**D**) shown for each MOI as indicated in the legends. Transient, non-productive viral infection detected as early as 48 h p.i. and as late as 144 h p.i. in mock condition (0 MOI), is likely the result of technical handling by the experimental operator.

The coronavirus HCoV-NL63 enters host cells through the same ACE2 receptor pathway as SARS-CoV-2^43^, and utilizes protein priming via the protease TMPRSS2, and therefore NL63 represents a useful model for studying SARS-CoV-2 infection in a BSL-2 laboratory environment. HCoV-OC43 and SARS-CoV-2 both belong to the betacoronavirus genus, and share common spike protein properties. In Fig. 9, we show infection kinetics determined by RT-qPCR for each of HCoV-NL63 (Fig. 9A,C) and HCoV-OC43 (Fig. 9B,D) across 12 d p.i. and across a range of MOIs, and we observe a strong increase in viral load versus MOI with distinct rates of infection for each strain. As discussed earlier, we believe that the positive detection of viral particles in some of the PREDICT96-ALI devices exposed to mock condition (0 MOI) was likely associated with user-introduced technical error or thresholding error.

### Treatment of HCoV-NL63 with camostat mesylate versus viral copy numbers

**Figure 10:**
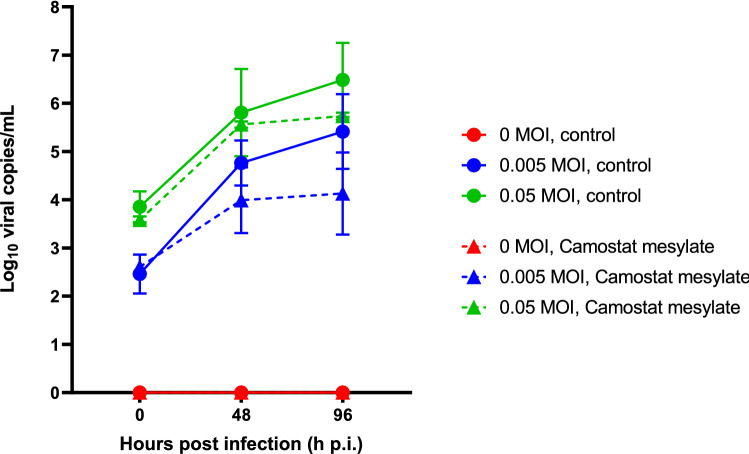
Efficacy of camostat mesylate against hCOV-NL63-inoculated PREDICT96-ALI airway tissue. RT-qPCR analyses for viral copies in the apical wash of PREDICT96-ALI airway tissue at 0, 48 and 96 h p.i. with HCoV-NL63 across multiple MOIs. Solid lines show viral copies for MOI 0 (red, mock control), MOI 0.005 (blue) and MOI 0.05 (green) when treated with vehicle (DMSO), and dashed lines show corresponding viral copies when treated with 20 µM camostat mesylate. N = 2–4 tissue replicates per donor, time-point and condition. Replicates that did not meet the minimum signal intensity after 45 PCR cycles are displayed along the x-axis.

Camostat mesylate (CM) is a serine protease inhibitor reported to partially block infection by HCoV-NL63 in Calu-3 cells^44^ and IAV in human primary bronchial epithelial cells^45^ and is under investigation as a treatment for SARS-CoV-2 in an ongoing clinical trial^46^. Here we evaluate the effect of CM administered to PREDICT96-ALI airway tissues cultured with human primary epithelial airway cells and inoculated with HCoV-NL63 across a range of MOIs (including mock control, 0 MOI). In Fig. 10, we show infection kinetics for the HCoV-NL63 strain across a range of MOIs for vehicle control versus 20 μM CM-treated cultures in PREDICT96-ALI. While the scatter limits statistical significance, we observe that CM treatment of airway tissues infected with HCoV-NL63 reduces viral copy number at both the 48 and 96 h time points, particularly for the MOI = 0.005 condition.

## Discussion

In this work, we leverage the PREDICT96-ALI platform to create a high-throughput airway infection model to assist in the development of therapeutics to treat respiratory viral infections. To do so, we developed a barrier tissue model at an ALI with human primary tracheobronchial cells, establishing a pseudostratified epithelium, and cultured atop a semipermeable membrane with independent flow control for each device in the bottom microchannel culture chamber. The data presented demonstrate a robust model for the human tracheobronchial airway comprising of ciliated, basal, club, and goblet cell populations in a physiologically representative distribution and epithelial layer architecture. Human primary epithelial airway cultures established using commercial sources of cadaver tissues were evaluated, as well as fresh bronchial epithelial cells obtained from healthy control subjects via research bronchoscopy. The latter approach represents a potential pathway toward in vitro investigation of respiratory infections as a function of gender, age and co-morbidities using tailored sources of patient specimens that would be difficult to access from commercial vendors.

This airway model was applied to the study of respiratory viral infections including IAV, HCoV-OC43 and HCoV-NL63, and infection kinetics were evaluated across multiple viral strains, donor populations, MOIs, and time points. Experiments conducted with multiple donor populations of NHBE cells suggest the potential of this system for conducting studies of donor-dependent mechanisms of infection and responses to therapeutic interventions. Key advantages of this platform over existing in vitro platforms include the use of human primary tracheobronchial epithelial cells cultured at an ALI, a more physiologically relevant cell-to-media ratio with smaller, more convenient cell culture area than current standard Transwell™ cultures, integrated pumping of basal media to maintain precision control over solute concentration and to avoid the development of non-physiological gradients, and compatibility with in situ, high resolution and real-time imaging. While certain existing organ-on-chip models possess some of these advantages, they are typically low-throughput systems designed for research environments, and are often incompatible with workflows and standard instrumentation in pharmaceutical laboratories. Considering the throughput limitations of the competing organ-on-chip models, the PREDICT96-ALI platform offers an unmatched capacity that enables large combinations of conditions, such as evaluation of three therapeutics across four MOIs and time points with two viral strains and four replicates for each condition simultaneously on a single plate. The PREDICT96-ALI platform combines the high throughput, precision control and physiological relevance necessary to serve as a powerful tool for applications in disease modeling, drug development, and screening. While the throughput of the 96-device platform with integrated pumping and automated, multiplexed TEER measurements represents an important step forward, further automation of the processes described herein would be necessary to establish a high efficiency workflow for pharmaceutical development.

A critically important need in the field of respiratory virus research is the establishment of robust in vitro disease models that provide the precision, validation, throughput, and utility necessary for routine operation in drug development laboratories. Until recently, in vitro models suitable for the study of respiratory viral infections have been either high-throughput but simplistic 2D culture plate-based systems utilizing cell lines, or low-throughput and complex organ-on-chip models that are difficult to accommodate in the workflow of many laboratories. The PREDICT96 platform has been reported previously, and represents the first high-throughput organ-on-a-chip platform with integrated precision flow control in a standard ANSI SLAS (American National Standards Institute/Society for Laboratory Automation and Screening) well plate format^28^. Here, we deployed the airway tissue model successfully in the PREDICT96-ALI platform to investigate infections of two pandemic strains of IAV (A/California/04/09 H1N1 and A/HongKong8/68 H3N2). Infections were monitored using a combination of IF and RT-qPCR, across a range of MOIs and time points, providing a strong foundation for evaluation of therapeutic interventions. As a proof of concept, oseltamivir dosing of the PREDICT96-ALI airway model was shown to reduce viral copies of A/HongKong8/68 H3N2 across two donors, demonstrating the potential of this system for screening therapeutic compounds for respiratory viruses. Finally, infection kinetics were established for human primary bronchial epithelial cells with two different human coronaviruses (HCoV-NL63 and HCoV-OC43), and treatment with the serine protease inhibitor camostat mesylate resulted in an observed reduction in viral copies, consistent with activity of CM against the TMPRSS2-mediated viral entry^41^.

Our human airway tissue model has applicability to the study of respiratory viruses including coronaviruses, and we have demonstrated the relevance of this model to monitoring pathogen infection kinetics. To investigate the relevance of our model to novel viral pathogens, like SARS-CoV-2, we examined the ability of HCoV-NL63, an alphacoronavirus sharing the same host entry receptor as SARS-CoV-2, to infect and replicate within PREDICT96-ALI. To do so, we first confirmed the presence of ACE2 and TMPRSS2 transcripts, as well as ACE2 protein expression, in the primary human cells within our model and successfully inoculated the PREDICT96-ALI airway model with HCoV-NL63 across a range of MOIs and for two different human donor primary epithelial cell populations. Expression of ACE2 and TMPRSS2 in various human tissues, and their relationship with mechanisms of coronavirus infections, remain a subject of intense study^47–51^. Evaluation of the relative expression of ACE2 and TMPRSS2 across tissue types and across donors represents a vitally important direction for further understanding mechanisms of COVID-19 infection. For instance, questions surrounding the occurrence of COVID-19-related anosmia^52^, and the reduced incidence of infection in young children^53^, have been associated with relative levels of ACE2 across nasal versus airway tissues and across various age groups. In a comprehensive study involving the Human Cell Atlas Lung Biological Network, ACE2 and TMPRSS2 expression across nasal, airway and lung tissues were evaluated, across age, sex and smoking status^54^. These studies not only shed light on critical questions related to COVID-19 pathogenesis, but also suggest avenues and strategies for therapeutic intervention. In our analysis, ACE2 and TMPRSS2 expression were evaluated across two donors and levels were relatively similar. Relative to the housekeeping gene GAPDH, the ACE2 levels we observed were of a similar order of magnitude as those reported elsewhere^55^, but expression is heavily dependent on several variables as described above. The number of recent lethal outbreaks of coronaviruses, SARS-CoV, MERS-CoV, and the on-going outbreak of SARS-CoV-2, highlight the need for human airway models that can be tailored to support and replicate a broad range of respiratory viruses and strains and enable screens for therapeutic treatment discovery. To demonstrate the utility and versatility of our model to support the study of multiple coronavirus genera and species, many of which target unique and sometimes unknown human host cell surface receptors, we directly compared inoculation of HCoV-NL63 with HCoV-OC43, a betacoronavirus targeting the 9-O-acetylated sialic acid receptor^56^. PREDICT96-ALI experiments explored the differences in infection kinetics between these two strains of coronavirus, indicating that the model is permissive to different coronavirus genera and facilitates replication of these viruses. In addition to the adaptability of the model, we also demonstrated the efficacy of a known antiviral agent in our high-throughput human primary cell-based airway platform, investigating the effect of the compound across various MOI in a single 96-device experiment. This new platform model for infection with coronaviruses represents an important new capability for studying coronavirus infections in human primary lung tissue and evaluating therapeutics, improving upon prior approaches that use immortalized cell lines in high-throughput systems^57–59^ or human primary lung cells in standard Transwell™ formats^29,60^.

In light of current challenges in identifying and developing therapeutic agents in response to emerging respiratory viral diseases, the ability to rapidly assess efficacy in a human relevant platform and to efficiently evaluate donor-to-donor variability via a high throughput system such as PREDICT96 is critical. These data demonstrate the promise of the PREDICT96-ALI airway model as a new capability for preclinical evaluation of therapeutics for respiratory infections including SARS-CoV-2 in an efficient, robust, and high-throughput manner. A key question relates to the suitability of this platform technology for operation in facilities required for experimentation with SARS-CoV-2 and other pathogens restricted to BSL-3/4 laboratory environments. The PREDICT96-ALI airway platform presents several critical features that render it suitable for use in high-containment facilities, including compact size and an absence of large off-chip laboratory pumps, as well as its ability to interface with standard laboratory infrastructure. These features allow for ease of handling of the system in personal protective equipment (PPE) such as powered air-purifying respirators (PAPR) and pressurized, full-body safety suits associated with high containment work. Further, the ability to adjust the precision controlled perfusion flow and to conduct biophysical sensing and detection in real-time, remotely (by way of Bluetooth), and multiplexed is critically important. Adaptation of the workflow and operational protocols for the PREDICT96-ALI system for use in high containment is underway, toward application of this platform for SARS-CoV-2 research.

## Supplementary Information

Supplementary Information 1.

Supplementary Video 1.

Supplementary Information 2.
