# Supplementary figures and images for: High-throughput human primary cell-based airway model for evaluating influenza, coronavirus, or other respiratory viruses in vitro
Source: Sci Rep. 2021 Jul 22;11:14961. doi: 10.1038/s41598-021-94095-7 (PMC8298517; doi:10.1038/s41598-021-94095-7)

## Slide 1
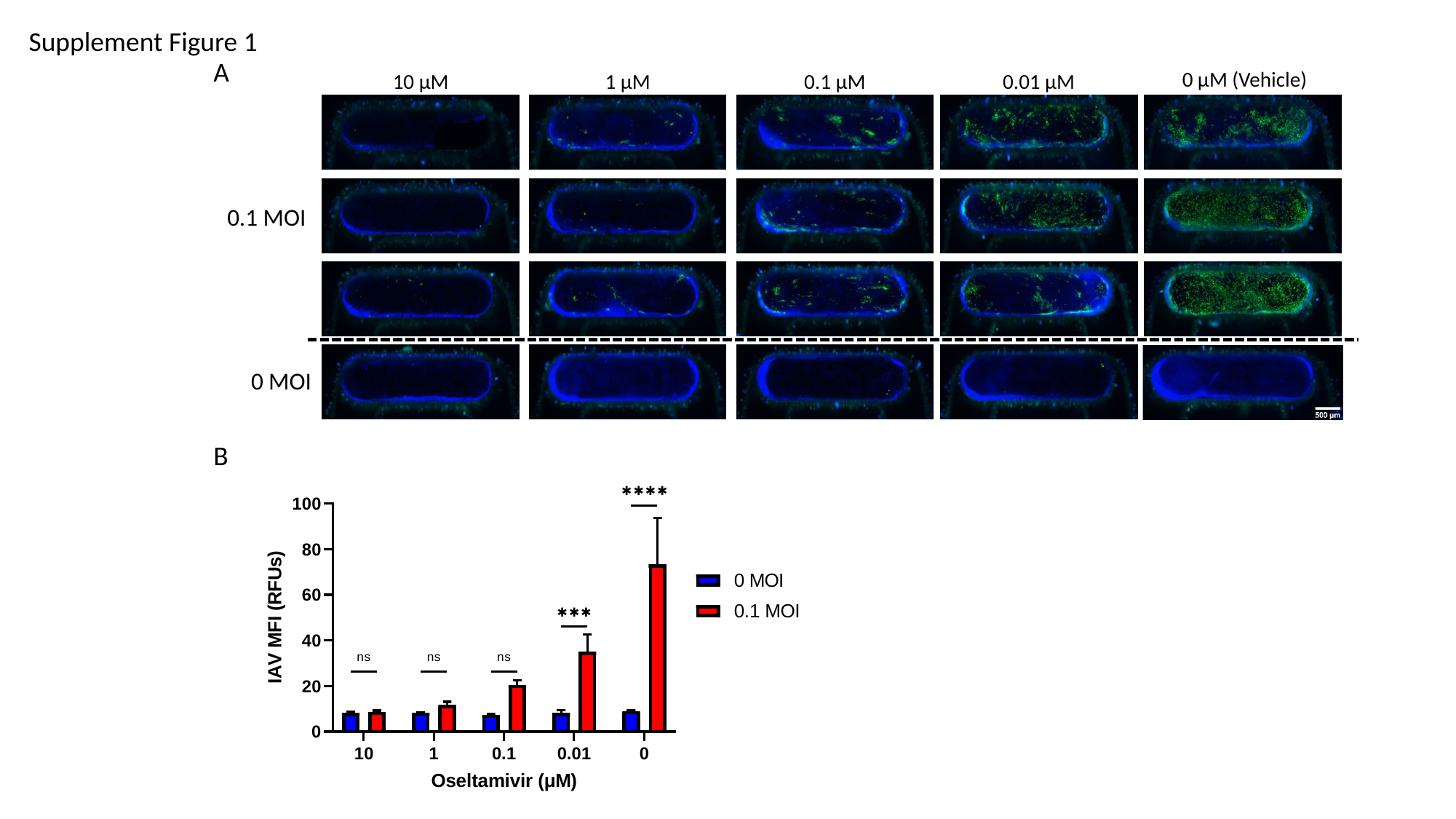

Supplement Figure 1
A
0 µM (Vehicle)
0.01 µM
10 µM
1 µM
0.1 µM
0.1 MOI
0 MOI
B

Supplement: Supplementary file 1 — Supplementary Information 1. [file 41598_2021_94095_MOESM1_ESM.pptx]
