# Supplementary material for: High-throughput human primary cell-based airway model for evaluating influenza, coronavirus, or other respiratory viruses in vitro
Source: Sci Rep. 2021 Jul 22;11:14961. doi: 10.1038/s41598-021-94095-7 (PMC8298517; doi:10.1038/s41598-021-94095-7)
Supplement: Supplementary file 3 — Supplementary Information 2. [file 41598_2021_94095_MOESM3_ESM.docx]

**Supplemental Figure 1. Quantification of IAV-inoculated PREDICT96-ALI Airway Tissue in Response to Oseltamivir. (a)** Viral infectivity (IAV-NP, green) of tissues treated with oseltamavir (Tamiflu) at 0, 0.01, 0.1, 1, or 10 µM and inoculated with A/Hong Kong/8/68 H3N2 at 0 (mock control) or 0.1 MOI was visualized and captured using a Celigo Image Cytometer following tissue following fixation and staining at 48 h p.i. **(b)** Quantification of the mean fluorescence intensity (MFI) of the IAV-NP stain (green) or Alexa Fluor 488 signal, indicating that oseltamivir doses >0.1uM significantly reduces IAV H3N2 infection at MOI of 0.1. Statistical significance: *** p ≤ 0.001, and **** p ≤ 0.0001. N = 2 independent experiments with data presented from Donor C as representative and from one representative experiment. Per experiment, N = 3 tissue replicates per donor, time-point and condition (MOI, anti-viral dose). The data presented corresponds to **Figure 6b**.

**Supplemental Video 1**. **Ciliary Beat in Mature PREDICT96-ALI Airway Tissues.** Ciliary beat of mature PREDICT96-ALI tissue grown to 28 d at an ALI. Videos were captured at 100 fps and at a magnification of 40x. Donor DH01 shown as representative.
